# Use of a Sparse-Response Deep Belief Network and Extreme Learning Machine to Discriminate Alzheimer's Disease, Mild Cognitive Impairment, and Normal Controls Based on Amyloid PET/MRI Images

**DOI:** 10.3389/fmed.2020.621204

**Published:** 2021-01-18

**Authors:** Ping Zhou, Shuqing Jiang, Lun Yu, Yabo Feng, Chuxin Chen, Fang Li, Yang Liu, Zhongxiong Huang

**Affiliations:** Department of PET-CT Center, Chenzhou No.1 People's Hospital, Chenzhou, China

**Keywords:** computer-aided diagnosis, Alzheimer's disease, mild cognitive impairment, sparse-response deep belief network, extreme learning machine

## Abstract

In recent years, interest has grown in using computer-aided diagnosis (CAD) for Alzheimer's disease (AD) and its prodromal stage, mild cognitive impairment (MCI). However, existing CAD technologies often overfit data and have poor generalizability. In this study, we proposed a sparse-response deep belief network (SR-DBN) model based on rate distortion (RD) theory and an extreme learning machine (ELM) model to distinguish AD, MCI, and normal controls (NC). We used [^18^F]-AV45 positron emission computed tomography (PET) and magnetic resonance imaging (MRI) images from 340 subjects enrolled in the ADNI database, including 116 AD, 82 MCI, and 142 NC subjects. The model was evaluated using five-fold cross-validation. In the whole model, fast principal component analysis (PCA) served as a dimension reduction algorithm. An SR-DBN extracted features from the images, and an ELM obtained the classification. Furthermore, to evaluate the effectiveness of our method, we performed comparative trials. In contrast experiment 1, the ELM was replaced by a support vector machine (SVM). Contrast experiment 2 adopted DBN without sparsity. Contrast experiment 3 consisted of fast PCA and an ELM. Contrast experiment 4 used a classic convolutional neural network (CNN) to classify AD. Accuracy, sensitivity, specificity, and area under the curve (AUC) were examined to validate the results. Our model achieved 91.68% accuracy, 95.47% sensitivity, 86.68% specificity, and an AUC of 0.87 separating between AD and NC groups; 87.25% accuracy, 79.74% sensitivity, 91.58% specificity, and an AUC of 0.79 separating MCI and NC groups; and 80.35% accuracy, 85.65% sensitivity, 72.98% specificity, and an AUC of 0.71 separating AD and MCI groups, which gave better classification than other models assessed.

## Introduction

Alzheimer's disease (AD) is a neurodegenerative disease characterized by cognitive dysfunction and associated with advanced age. Because there are currently no therapies that can reverse the course of AD, it is important to diagnose AD and its prodromal stage, mild cognitive impairment (MCI) as early as possible (1).

In recent years, neuroimaging techniques have been shown to be effective tools for the diagnosis of AD. Magnetic resonance imaging (MRI) and positron emission tomography (PET) are two common neuroimaging methods. For example, Hua et al. proposed a powerful tool to monitor structural atrophy in incipient stages of AD using MR images (2). Mosconi et al. demonstrated that PET scans may provide objective and sensitive support to clinical diagnosis in early dementia (3). In addition, deep learning methods have shown great promise for image analysis and disease prediction. For instance, Hu et al. utilized a targeted autoencoder network to classify functional connectivity matrices across brain regions, which was able to distinguish MCI from NC with 87.5% accuracy (4). Liu et al. designed a deep learning architecture to more accurately differentiate AD, MCI, and normal controls (NC). The architecture, including stacked autoencoders and a softmax output layer, achieved 87.76% accuracy, 88.57% sensitivity, and 87.22% specificity distinguishing AD from NC and exhibited 76.92% accuracy, 74.29% sensitivity, and 78.13% specificity distinguishing MCI from NC (5). In addition, a few of deep learning studies based on PET/MRI could also be observed (6, 7).

However, the methods mentioned above had some disadvantages. For instance, gradient diffusion and gradient explosion may emerge with deepening of the autoencoder stack depth, resulting in decreased classification accuracy. To mitigate this limitation, we proposed a sparse-response deep belief network (SR-DBN) based on the rate distortion (RD) theory model. Our SR-DBN used the contrastive divergence algorithm to maximize the retention of data distribution, in case gradient diffusion and gradient explosion became factors. In addition, the SR-DBN model included sparsity. Compared to DBN models without sparsity, sparse representations allow changing the significant bits for each example in a fixed-size representation, which are more efficient from the point of view of information theory (8). Subsequently, we used an extreme learning machine (ELM) as a classifier to get the performance of the classification. Meanwhile, to evaluate the effectiveness of our method, we compared our model with other models.

## Model Design

### Model Framework

**Figure 1:**
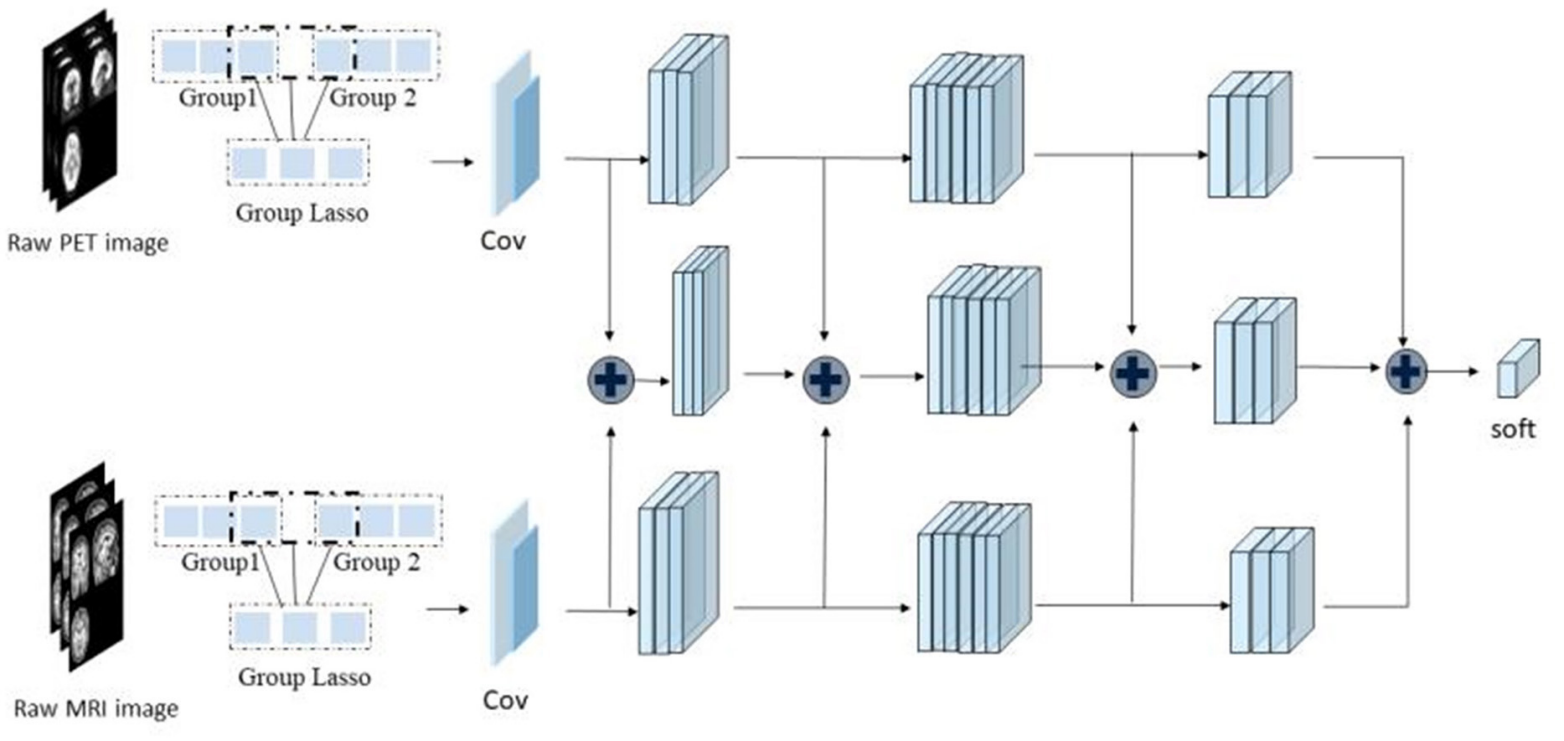
The framework of this study.

As shown in Figure 1, the framework of the model consists of four parts: (1) original image data underwent standard preprocessing; (2) data dimensionality was reduced using fast principal component analysis (PCA); (3) features were extracted by three SR-DBNs based on rate distortion theory; and (4) processed data were classified by the ELM.

### Mathematical Fundamentals of the Proposed Model

#### SR-DBN Model Based on RD Theory

Restricted Boltzmann machines (RBM) are neural perceptrons composed of a visible layer and a hidden layer. Several RBMs can form a DBN. Similar to the structure of a DBN, the SR-DBN also consists of several sparse-response restricted Boltzmann machines (SR-RBMs). In the model, the Kullback–Leibler divergence *KL*($p^{0}∥p_{\theta }^{\infty }$) (9) between the original data's distribution *p*^0^ and the equilibrium distribution $p_{\theta }^{\infty }$ defined by RBM served as a distortion function. Considering the RD theory, we can deduce the following formulation:

(1) $$\begin{array}{l} \underset{w_{ij},c_{i},b_{j}}{\text{min}}KLp^{0}∥p_{\theta }^{\infty }+\lambda \overset{m}{\underset{l=1}{\sum }}{∥p\left({\text{h}}^{\left(\text{l}\right)}|{\text{v}}^{\left(\text{l}\right)}\right)∥}_{1} \end{array}$$

where $\overset{m}{\underset{l=1}{\sum }}{∥p\left({\text{h}}^{\left(\text{l}\right)}|{\text{v}}^{\left(\text{l}\right)}\right)∥}_{1}$ denotes the sparseness of representation and λ is a regularization parameter. Then we replaced Kullback–Leibler divergence with $p^{0}||p_{\theta }^{\infty }-p_{\theta }^{1}||p_{\theta }^{\infty }$ to simplify calculations (10). Suppose w is the weight matrix of RBM, b is the bias vector of the input layer, and c is the bias vector of the output layer, giving the updated rules below:

(2) $$\begin{array}{l} \{\begin{array}{c} w_{ij}:=w_{ij}+\epsilon \left({\langle v_{i}h_{j}\rangle }_{p^{0}}-{\langle v_{i}h_{j}\rangle }_{p_{\theta }^{1}}\right)\\ c_{i}:=c_{i}+\epsilon \left({\langle v_{i}\rangle }_{p^{0}}-{\langle v_{i}\rangle }_{p_{\theta }^{1}}\right)\\ b_{j}=b_{j}+\epsilon \left({\langle h_{j}\rangle }_{p^{0}}-{\langle h_{j}\rangle }_{p_{\theta }^{1}}\right) \end{array} \end{array}$$

where ϵ denotes a learning rate. Additionally, we added another update with the gradient of the regularization term in each iteration. The term is as follows:

(3) $$\begin{array}{l} -\frac{\partial }{\partial w_{ij}}\overset{m}{\underset{l=1}{\sum }}{∥p\left({\text{h}}^{\left(\text{l}\right)}|{\text{v}}^{\left(\text{l}\right)}\right)∥}_{1}=-\overset{m}{\underset{l=1}{\sum }}\frac{\partial }{\partial w_{ij}}\mathrm{sigmoid}\left(b_{j}+\underset{i}{\sum }w_{ij}\nu_{i}^{\left(l\right)}\right)\\ \;=-\overset{m}{\underset{l=1}{\sum }}p_{j}^{\left(l\right)}\left(1-p_{j}^{\left(l\right)}\right)\nu_{i}^{\left(l\right)} \end{array}$$

(4) $$\begin{array}{l} -\frac{\partial }{\partial b_{j}}\overset{m}{\underset{l=1}{\sum }}{∥p\left({\text{h}}^{\left(\text{l}\right)}|{\text{v}}^{\left(\text{l}\right)}\right)∥}_{1}=-\overset{m}{\underset{l=1}{\sum }}\frac{\partial }{\partial w_{ij}}\mathrm{sigmoid}\left(b_{j}+\underset{i}{\sum }b_{j}\nu_{i}^{\left(l\right)}\right)\\ \;=-\overset{m}{\underset{l=1}{\sum }}p_{j}^{\left(l\right)}\left(1-p_{j}^{\left(l\right)}\right) \end{array}$$

where $p_{j}^{\left(l\right)}=\text{sigmoid}\left(\sum_{i}\nu_{i}^{\left(l\right)}w_{ij}+b_{j}\right)$, and sigmoid(.) represents the sigmoid function.

In this study, we employed one input layer, three hidden layers and one output layer.

#### ELM Model for Classification

An ELM is a neural network algorithm for a single hidden layer feedforward neural network. Its input weights and hidn node bias are generated randomly within a given range. The only optimal solution can be obtained by setting the number of hidden layer neurons (11). When the input weights and hidden layer bias are determined randomly, the output matrix of the hidden layer, H is also determined (12):

(5) $$\begin{array}{l} \hat{\beta }=H^{+}T \end{array}$$

where H^+^ is the Moore–Penrose pseudoinverse matrix of H and the T notes the expected output.

## Materials and Methods

### Materials

The data used in this study were access through the Alzheimer's Disease Neuroimaging Initiative (ADNI) public database. ADNI is a consortium study initiated in 2004 by the National Institute on Aging, the National Institute of Biomedical Imaging and Bioengineering, the Food and Drug Administration, private pharmaceutical companies, and nonprofit organizations (13). For additional information about ADNI, please see www.adni-info.org.

In this study, we selected AV45 PET and structural MRI images of 340 subjects enrolled in ADNI, including 116 AD, 82 MCI, and 142 NC subjects. The clinical data for each of these diagnostic groups is shown in Table 1.

**Table 1:** The clinical data of three cohorts.

	**AD (*n* = 116)**	**MCI (*n* = 82)**	**NC (*n* = 142)**
Gender (M/F)	68/48	50/32	52/90
Ages (years)	75.37 ± 5.61	75.6 ± 5.87	73.86 ± 7.06
MMSE	21.63 ± 3.97	23.8 ± 5.98	28.92 ± 1.27
MOCA	15.89 ± 5.85	18.8 ± 6.49	26.01 ± 2.76

### Image Preprocessing

MRI data were acquired on multiple 3T MRI scanners using scanner-specific T1-weighted sagittal 3D MPRAGE sequences. In order to increase signal uniformity across the multicenter scanner platforms, original MPRAGE acquisitions underwent standardized image preprocessing steps. The current study implemented the following steps: (1) segmentation of the images into gray matter (GM), white matter (WM) and cerebrospinal fluid (14), of which gray matter and white matter were used for further analysis; (2) normalization of all GM and WM images into Montreal Neurological Institute space; and (3) spatial smoothing using a Gaussian kernel of 4 mm^3^.

[18F]-AV45 PET data were acquired on multiple instruments of varying resolutions and following different platform-specific acquisition protocols. Similar to the MRI data, ADNI PET data underwent standardized image preprocessing steps aimed at increasing data uniformity across the multicenter acquisitions (15). The preprocessing steps included realignment, spatial normalization to MNI space, and smoothing using a 7-mm^3^ Gaussian kernel. We performed a voxel-based partial volume effects correction of the normalized functional image using the Müller-Gärtner method. Lastly, the partial volume effect-corrected functional image was smoothed to reduce noise and improve image quality using an isotropic Gaussian smoothing kernel with a full width at half maximum setting of 7 mm^3^. The image was scaled up to obtain a standard uptake value rate map of the entire cerebellum.

Both MRI and ^18^F-AV45 PET images were preprocessed using statistical parametric mapping software (SPM12, https://www.fil.ion.ucl.ac.uk/spm/software/spm12/) on Matlab 2016b.

### Dimension Reduction and Feature Extraction

Fast PCA was used to describe the data with a small number of linearly independent features under the principle of ensuring the minimum loss of data information.

In the study, the SR-DBN model undertook feature extraction. Compared with DBN, the SR-DBN is more efficient from the perspective of information theory, which allows changing the effective number of bits per example in a fixed size representation (8).

The SR-DBN model used in the study was made up of multiple basic SR-RBMs with the same numbers of nodes. The output of each SR-RBM was the input of the next basic SR-RBM at successive levels. In the last layer of the SR-DBN model, a back propagation network was set, receiving the output feature vector of SR-RBM as learned features, and adopting a gradient descent algorithm to fine-tune the weight of the whole network, thereby coordinating and optimizing the parameters of the whole SR-DBN.

### Classification & Comparative Experiments

Three kinds of images were used as input: [^18^F]-AV45 PET, and GM and WM segmentations from the MRI. Correspondingly, we used three SR-DBNs to extract features. Following feature extraction, the ELM classified the three diagnostic groups. After obtaining the predicted labels, the accuracy, sensitivity, specificity, and area under curve (AUC) were calculated to evaluate the practicability of the model.

The model was evaluated using five-fold cross-validation, repeated 200 times. In the case of “lucky trails,” we randomly sampled the training and testing instances from each class to ensure they had similar distributions as the original dataset. The entire network was trained and fine-tuned with 80% of the data and then tested with the remaining 20% of the samples in each validation trial.

To evaluate the effectiveness of our method, we performed several comparative trials. In contrast experiment 1, ELM was replaced by a support vector machine (SVM). Contrast experiment 2 utilized DBN without sparsity. Contrast experiment 3 consisted of fast PCA and ELM. Contrast experiment 4 used a classic convolutional neural network (CNN) to classify AD, MCI, and NC. The experimental platform is based on Matlab 2016b.

## Results

### Results of Dimension Reduction

**Figure 2:**
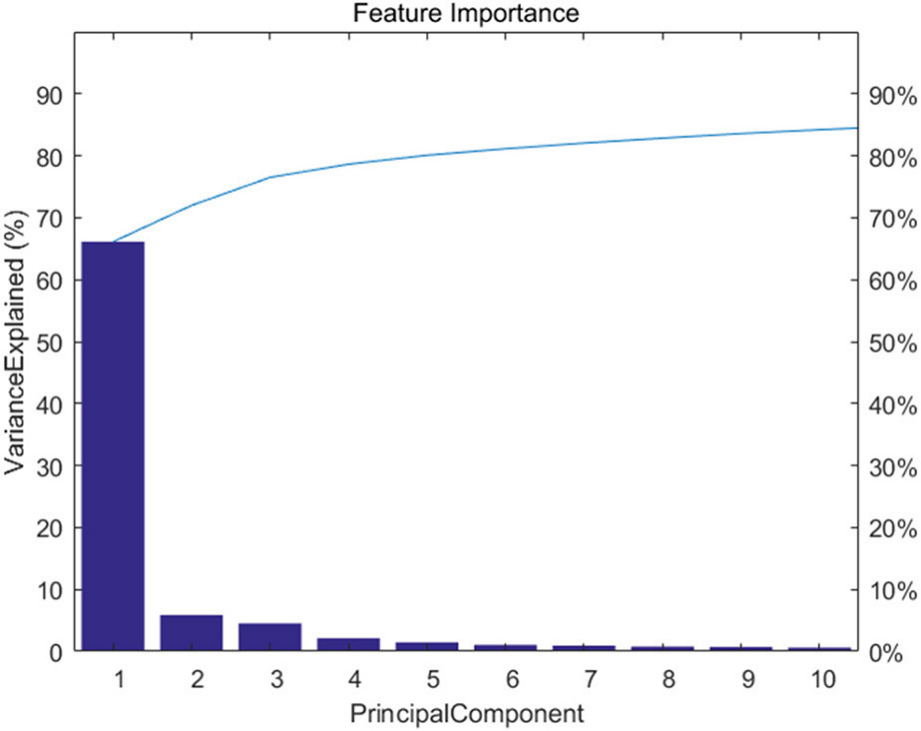
The feature importance of fast PCA.

Figure 2 shows feature importance. We extracted the top 20 features which represent 90% information of the original data.

### Results of Classification

**Figure 3:**
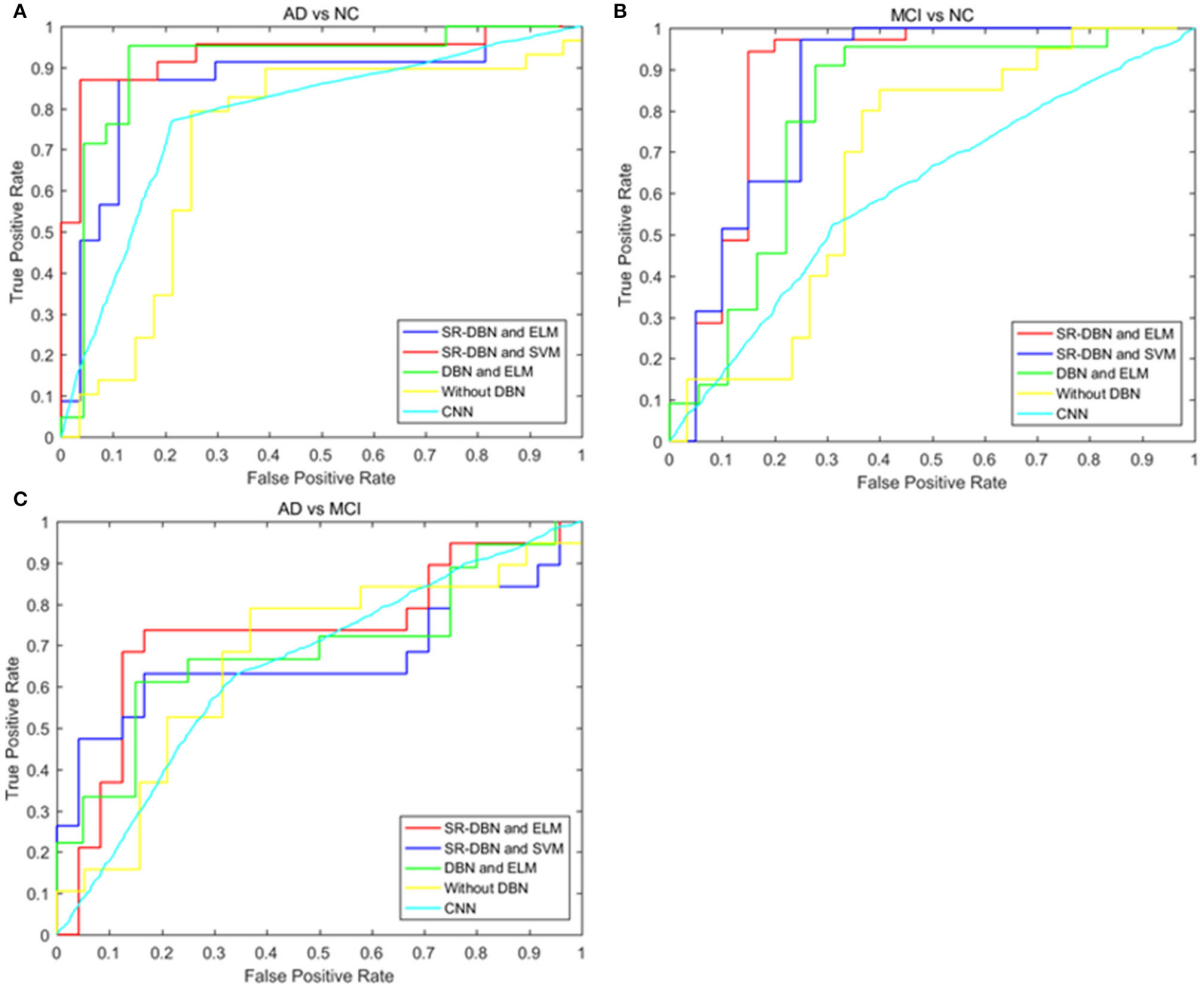
The ROC curves of five models in the three experiments. **(A)** Shows the ROC curves of the classification of AD and NC; **(B)** shows the ROC curves of the classification of MCI and NC; **(C)** shows the ROC curves of the classification of AD and MCI.

The classification and comparative results are shown in Table 2 and Figure 3. In the classification of AD and NC, our model achieved 91.68% accuracy, 95.47% sensitivity, 86.68% specificity, and an AUC of 0.87. In the classification between MCI and NC, the model achieved 87.25% accuracy, 79.74% sensitivity, 91.58% specificity, and an AUC of 0.79. When separating between AD and MCI, the model achieved 80.35% accuracy, 85.65% sensitivity, 72.98% specificity, and an AUC of 0.71. Moreover, the time cost for image processing and classification in our proposed method and four compared methods were 36.2 s, 37.4 s, 491.7 s, 25 s, and 1,386.5 s. This result means that our method is faster than the classical CNN model, and similar to machine learning models.

**Table 2:** Results of the experiment.

		**Proposed**	**Experiment 1**	**Experiment 2**	**Experiment 3**	**Experiment 4**
AD vs. NC	ACC (%)	91.68 ± 1.09	85.68 ± 1.6	86.28 ± 1.02	76.43 ± 0.5	77.12 ± 0.04
	SEN (%)	95.47 ± 1.73	90.58 ± 2.43	90.92 ± 1.19	80.01 ± 0.62	74.02 ± 0.08
	SPE (%)	86.68 ± 2.61	79.6 ± 3.81	80.31 ± 2.24	71.99 ± 0.76	79.53 ± 0.13
	AUC	0.87 ± 0.01	0.82 ± 0.01	0.83 ± 0.02	0.75 ± 0.02	0.77 ± 0.03
MCIvsNC	ACC (%)	88.25 ± 1.38	80.34 ± 1.79	80.18 ± 1.03	67.64 ± 0.54	63.15 ± 0.02
	SEN (%)	79.74 ± 3.44	68.85 ± 4.77	68.33 ± 3.16	45.72 ± 1.1	78 ± 0.09
	SPE (%)	91.58 ± 2.14	87.19 ± 2.73	87.22 ± 1.55	80.05 ± 0.53	41.96 ± 0.11
	AUC	0.79 ± 0.01	0.72 ± 0.03	0.73 ± 0.03	0.60 ± 0.04	0.60 ± 0.02
ADvs MCI	ACC (%)	80.35 ± 1.8	73.07 ± 2.52	71.95 ± 1.61	65.77 ± 0.6	63.71 ± 0.03
	SEN (%)	85.65 ± 3.63	76.3 ± 3.93	78.51 ± 2.42	74.49 ± 0.72	79.44 ± 0.08
	SPE (%)	72.98 ± 4.71	69.41 ± 5.79	63.33 ± 4.06	54.15 ± 1.07	41.27 ± 0.09
	AUC	0.71 ± 0.08	0.69 ± 0.01	0.68 ± 0.02	0.59 ± 0.04	0.6 ± 0.02

Table 3 shows the comparative results of our model and results from the literature, including Hu's model, Liu's model, and Suk's model (4, 5, 16). Specifically, Hu's model used a single image modality (MRI) and Liu's model used both MRI and PET. Our proposed model achieved the best classification result of all models compared.

**Table 3:** Comparison of classification performances (%).

	**AD vs. NC**			**MCI vs. NC**		
	**ACC (%)**	**SEN (%)**	**SPE (%)**	**ACC (%)**	**SEN (%)**	**SPE (%)**
Our model	91.68	95.47	86.68	88.25	79.74	91.58
Hu's model	-	-	-	87.5	-	-
Liu's model	87.76	88.57	87.22	76.92	74.29	78.13
Suk's model	83.2	-	-	70.1	-	-

## Discussion

In this paper, we used a SR-DBN and ELM for the classification of AD, MCI, and CN. In Table 2 and Figure 3, the superiority of our model compared to other models can be seen, as evidenced by the highest values for accuracy, sensitivity, specificity, and AUC.

Table 3 presents a comparison of our model with previous deep learning models from the literature. Hu's model, Liu's model, and Suk's model adopted the stacked autoencoders and softmax classifier to classify AD. The thickness of the method likely contributed to gradient diffusion and gradient explosion, which was successfully avoided by using CD in our model. In addition, Hu's model used a single imaging modality (MRI) and Liu's model was a multimodal example. As shown in Table 3, the performance of our model was superior to the two models, reflecting the potential utility of our model to aid in early AD diagnosis.

However, the study has several limitations. Firstly, the parameters of the model ought to be modified to obtain better performance. Secondly, the method is based on multimodal data, but subjects with missing image data points are excluded, limiting the sample size. Thirdly, we only compared classification results among our proposed SR-DBN model, machine learning models, and classical CNN models in our dataset. Other deep learning models, such as recurrent neural networks, and deep neural network models were not compared in the same dataset. They will be implemented and compared in the future. Finally, the data used were from Western patients, which could potentially affect the results. Data from Eastern patients should be included in future studies to optimize the model and make it more generalizable to Eastern populations.

## Conclusion

In the study, we proposed a SR-DBN combined with ELM to classify AD, MCI, and NC. Our model achieved 91.68% accuracy, 95.47% sensitivity, 86.68% specificity, and an AUC of 0.87 on the classification between AD and NC participants; 87.25% accuracy, 79.74% sensitivity, 91.58% specificity, and an AUC of 0.79 on classification between MCI and NC participants; and 80.35% accuracy, 85.65% sensitivity, 72.98% specificity, and an AUC of 0.71 on the classification between AD and MCI patients. Our model obtained better classification compared other models examined, indicating its effectiveness.

## Data Availability Statement

Publicly available datasets were analyzed in this study. This data can be found here: the Alzheimer's Disease Neuroimaging Initiative (ADNI) public database.

## Author Contributions

PZ, SJ, and LY are responsible for writing experimental procedures, organizing experimental results and writing the paper. YF, CC, FL, and YL are responsible for experimental data collection and preprocessing. ZH was responsible for proposing experimental plans and guiding the writing of the paper. All authors contributed to the article and approved the submitted version.

## Conflict of Interest

The authors declare that the research was conducted in the absence of any commercial or financial relationships that could be construed as a potential conflict of interest.

## Abbreviations

AD: Alzheimer's disease
ADNI: Alzheimer's disease neuroimaging initiative
AUC: area under curve
CAD: computer-aided diagnosis
CNN: convolutional neural network
ELM: extreme learning machine
GM: gray matter
MCI: mild cognitive impairment
MRI: magnetic resonance imaging
NC: normal controls
PCA: principal components analysis
PET: positron emission computed tomography
RBM: restricted Boltzmann machine
RD: rate distortion
SR-DBN: sparse-response deep belief network
SVM: support vector machine
WM: white matter.
